# Effects of Regular Long-Term Circuit Training (Once per Week) on Cardiorespiratory Fitness in Previously Sedentary Adults

**DOI:** 10.3390/ijerph182010897

**Published:** 2021-10-17

**Authors:** Verena Menz, Hannes Gatterer, Sachin B. Amin, Reinhard Huber, Martin Burtscher

**Affiliations:** 1 Department of Sport Science, University of Innsbruck, 6020 Innsbruck, Tyrol, Austria; Sachin.Amin@uibk.ac.at (S.B.A.); Martin.Burtscher@uibk.ac.at (M.B.); 2 Institute of Mountain Emergency Medicine, EURAC Research, 39100 Bolzano, Italy; Hannes.Gatterer@eurac.edu; 3 Sporttherapie Huber und Mair, Neu-Rum, 6020 Innsbruck, Tyrol, Austria; reini@sporttherapie-hm.at

**Keywords:** body weight training, untrained individuals, maximal oxygen consumption, ageing, physical activity intervention, real-life training intervention

## Abstract

The purpose of the study was (1) to investigate the effects of regular long-term circuit training (once per week) on cardiorespiratory fitness (CRF) in sedentary adults and (2) to compare training progress with the effects of continued exercise participation by regularly active age-matched individuals. Ten sedentary, middle-aged (51 ± 6 years) individuals (sedentary group, SG) of both sexes performed 32 weeks (1 training session/week) of supervised circuit training and 10 weeks of self-managed training. Effects were compared to an age-matched group (51 ± 8 years; *n* = 10) of regularly active individuals (active group, AG). CRF (expressed as peak oxygen uptake: VO_2_peak; peak power output: PPO) and systemic blood pressure (BP) during the incremental test were measured at the start and after the training intervention. CRF decreased significantly within the AG (VO_2_peak: 43.1 ± 7.3 vs. 40.3 ± 6.5 mL/min/kg, *p* < 0.05; PPO: 3.3 ± 0.6 vs. 3.1 ± 0.6; *p* < 0.05) but was maintained in the SG. In addition, significant improvements in restoration of the oxygen level in leg muscles after exercise and reduced systolic BP (180 ± 14 vs. 170 ± 17 mmHg, *p* = 0.01) at submaximal exercise were found within the SG. However, differences in changes from pre to post did not reach significance between groups. In contrast to the regularly active individuals, circuit training once per week over 32 weeks prevented the aging-related decline of CRF in previously sedentary subjects and reduced systolic BP during submaximal exercise, indicating improved exercise tolerance.

## 1. Introduction

Regular physical activity may be considered as the most important condition counteracting metabolic and cardiovascular diseases [1]. Furthermore, higher levels of activity are strongly associated with reduced risk of mortality in individuals with and without cardiovascular disease [2,3]. Several studies report that physical activity is consistently correlated with individual fitness level in a dose response manner. Typically, physical fitness is expressed as cardiorespiratory fitness (CRF), determined by incremental exercise testing [4] and expressed as peak oxygen consumption (VO_2_peak) and/or peak power output (PPO). Kokkinos et al. [3] demonstrated an approximately 20% lower mortality risk for subjects with an exercise capacity of 5–7 metabolic equivalents (METs; 1 MET = 3.5 mL O_2_∙min^−^^1^∙kg^−^^1^) compared to those achieving below 5 METs. This risk was reduced by 50% with an exercise capacity of 7–10 METs and even by 70% in those achieving more than 10 METs. These findings impressively point out that low CRF represents an independent risk factor for all-cause mortality and cardiovascular disease mortality, as well as providing multiple additional health benefits [5,6]. Aerobic and resistance exercises are the core modalities of exercise programs, particularly for older adults, as outlined by The American College of Sports Medicine (ACSM) [7]. Both modalities can considerably contribute to CRF and other health-related factors [8]. Endurance training especially results in reduced resting heart rate, blood pressure, and fat mass, while resistance training primarily increases muscle strength and power, bone mineral density and basal metabolism [8]. Typically, endurance training is performed as running, cycling, or swimming [9]. However, in recent times, functional/circuit training, mostly executed with the own body weight and with no or minimal equipment, has gained in popularity as it offers more variability of the training, combining aerobic and resistance exercises, and may therefore be perceived as less boring than classical endurance training. It has been shown that this kind of training induces aerobic improvement to the same extent as traditional endurance training [9,10]. This is highlighted by a recent review with meta-analysis, demonstrating the positive effect of circuit resistance training on CRF in middle-aged and older women [11] Moreover, this is also underlined by the study of Myers et al. [12], demonstrating that a circuit-based training with body-weight exercises elicited even greater improvements than a traditional training program. Moreover, both low resistance circuit and endurance training (over 12 weeks, 3 days per week) effectively improved aerobic capacity in sedentary adults [13].

According to international physical activity guidelines, adults should accumulate at least 150 min of moderate-to-vigorous-intensity physical activity per week [14,15,16], including both aerobic exercise and resistance training [7] for promoting health and improving CRF. Several studies on sedentary and untrained populations with three supervised training sessions/week showed the beneficial influence of physical activity on CRF, body composition, and quality of life [12,17,18]. However, the transfer of the published results into real life turns out to be challenging, as a large proportion of people fail to meet the above-mentioned recommendation of 150 min of physical activity [12,19,20]. More concerning is the fact that approximately 50% of adults fail to perform at least one bout of exercise per week [12]. 

Therefore, as current published guidelines are clearly not being met by the majority of the population, the question arises whether once-weekly supervised training as may be performed by individuals starting to engage in physical activity, would be a sufficient enough stimulus to improve CRF. One supervised training session per week may encourage persons to engage in unsupervised self-training, which may help to meet the exercise recommendation of 150 min. Bacon et al. [21] established that sedentary individuals with low physical fitness would demonstrate modest improvements in CRF even when performing a level of exercise far below the weekly recommendations. In addition, Warburton and Bredin [15] questioned the dose of exercise necessary for promoting health and concluded that 75 min per week of moderate- to vigorous-intensity physical activity may be sufficient to generate health benefits. 

The association between CRF and the development of risk factors is well known, but the time course for the preventive or therapeutic effectiveness of physical activity as well as the effects of “real-life” training interventions with just one supervised training session per week remain elusive. Thus, the main aim of this study was (1) to evaluate the effects of regular long-term circuit training (exercise training mostly executed with the own body weight or low equipment over 32 weeks) on CRF in sedentary adults and (2) to compare training progress with the effects of continued exercise participation by regularly active age-matched individuals. We hypothesized that one year of supervised, once weekly circuit training in previously sedentary individuals would be sufficient to improve CRF, eventually reaching values comparable to those of their already active counterparts.

## 2. Materials and Methods

### 2.1. Participants

Fifteen middle-aged individuals (39–65 years) of both sexes who participated in a regular and supervised physical activity program (once weekly circuit training) over 6 years (AG, active group) were recruited from a local training center. Fifteen age- and sex-matched sedentary persons (not having engaged in regular routine exercise 12 months prior to the study; not regularly performing any kind of exercise) (SG, sedentary group) were recruited via fliers and advertisement for the study. Possible exclusion criteria were BMI ≥ 30 kg/m^2^, age younger than 30 years or older than 65 years, musculoskeletal abnormality that would limit exercise participation, as well as all types of acute and chronic diseases. Prior to acceptance to the study, participants had to complete questionnaires regarding medical history and physical activity. During the study period, one female and four male participants of the SG as well as two female and three male participants of the AG had to withdraw due to time constrains or illness/injury. Finally, 10 participants per group completed the study and were incorporated into the current dataset. The study was carried out according to the Declaration of Helsinki and was approved by the Institutional Review Board of the Department of Sport Science, University Innsbruck (Certificate of good standing 08/2016; 29 February 2016). All participants gave written informed consent to participate in the study. Group-specific baseline characteristics of the participants are presented in Table 1.

### 2.2. Measurements

**Figure 1:**
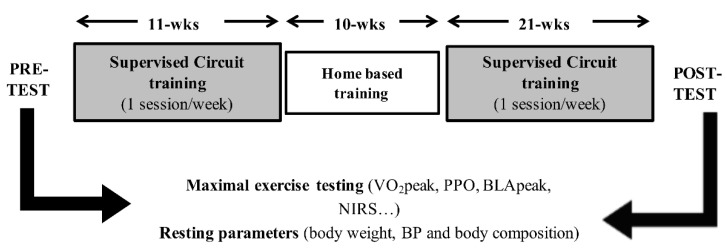
Timeline and main measurements of the study. BLApeak, peak blood lactate concentration; BP, blood pressure; VO_2_peak, peak oxygen uptake; PPO, peak power output; NIRS, near infrared spectroscopy; wks, weeks.

Laboratory assessments and performance testing were performed at the start and after the training period (Figure 1). 

#### 2.2.1. Resting Measurements

Resting heart rate (HR) and systemic blood pressure (Omron M4 Plus, Mannheim, Germany) were measured after resting for five minutes in a sitting position. Body mass (to the nearest 0.1 kg) and body composition were measured prior to the exercise test. Prior to the measurements, participants were instructed to empty their bladder. Body composition was determined by bioelectrical impedance analysis (BIA 101 Anniversary AKERN/RJL Systems; Florence, Italy), including the measurement of fat mass (FM), fat free mass (FFM), and muscle mass (MM). 

#### 2.2.2. Exercise Testing

Incremental cycling tests with gas analysis were performed to determine CRF (VO_2_peak and PPO). Tests were performed in the late morning at least 1 h after breakfast. No intense physical activity was permitted for the 3 days prior to the tests. The starting workload was 25 watts for females and 50 watts for males, which was increased by 25 watts every 2 min for females and 50 watts every 3 min for males until participants were unable to continue because of pain, fatigue, or dyspnea. Blood lactate concentrations (BLA; Biosen C line, EKF Diagnostics, Germany) from the hyperaemized ear lobe and ratings of perceived exertion (RPE) according to the ‘‘Borg scale’’ (6–20) [22] were documented at the end of each workload. Submaximal values from the incremental test were obtained at 75 watts for females (except for one female at 50 watts) and 100 watts for males. Gas analysis was performed using an open spirometric system (Oxycon Pro, CareFusion GmbH, Hoechbach, Germany), which was calibrated before each measurement, according to the manufacturer’s guidelines. Ventilatory parameters were recorded breath by breath during the ergospirometry. The HR was determined by a chest belt (Wear Link, Polar, Kempele, Finland) and transmitted to the spirometric device. VO_2_peak was defined as the highest 30 s average in oxygen uptake and peak heart rate (HRpeak) as the highest 10 s average during the test. Systolic (SBP) and diastolic blood pressure (DBP) were recorded in resting conditions and at 100 watts during the incremental test. 

Changes in muscle oxygenation were recorded using near infrared spectroscopy (NIRS; Niro 200, Hamamatsu Photonics K.K., Hamamatsu City, Japan) during the incremental test for evaluating quantitative blood flow and tissue oxygenation status [23]. Tissue oxygenation index (TOI = rSO_2_) was chosen as the master index for muscle oxygenation. The NIRS probe was longitudinally placed on the lateral part of musculus vastus lateralis on the left leg. Muscle deoxygenation during the incremental test was calculated by taking the lowest TOI value reached during the test minus the 1-min average TOI before the start of the test. Muscle reoxygenation was calculated while subtracting the 1-min TOI average after the test from the lowest TOI values during the incremental test. Changes in normalized tissue hemoglobin index (nTHI; percentage change in the amount of initial hemoglobin) were analyzed using the mean value of the entire incremental test and the last 30 s of the test. nTHI was expressed relative to the resting value. 

### 2.3. Intervention, Physical Activity

The AG maintained their regular training workload over the course of the study. This included one supervised 75 min circuit training session (similar circuit exercises as the SG) and their self-managed training outside the study (predominantly running, cycling, fitness training, hiking, alpine skiing, and ski mountaineering). 

Supervised circuit training for the SG begun at a low intensity/with low impact and was progressively increased. Total supervised training time per week was 75 min. The routines consisted of a 15 min warm-up of light aerobic and mobilization exercise, followed by circuit training (6–10 stations; 10–90 s exercise time) focusing on endurance and strength and a cool-down of 10 min. The training dose (duration × intensity) of the supervised circuit exercise sessions was increased in a stepwise manner to guarantee sufficient training stimulus to match the training dose performed by the AG in their supervised circuit training session. 

The routines were always modified to sustain interest in the training regime. Regarding the SG, the first few weeks of training focused on building core strength and improving leg axis stability. Training then progressed towards increasing whole body strength and endurance. Sessions ended with high-intensity exercises separated by short periods of recovery. Training was predominantly performed using body weight exercises and/or with small equipment (e.g., kettlebells, skipping rope, stability balls). Participants trained once a week in the late evening in a group of 10 to 15 persons, supervised by a sports scientist. The training intervention was split into 32 weeks of supervised training and 10 weeks of home-based, self-managed training as we followed the general course program where non-supervised training is provided during the summer holidays (Figure 1). The self-managed training was not controlled. 

### 2.4. Statistical Analysis

Normal distribution of variables was assessed with a Kolmogorov–Smirnov-test. Data are presented as mean ± standard deviation (SD). A mixed analysis of variance (ANOVA) measurement design was used to determine changes due to the training intervention (main effect: time) and to determine different changes between the AG and SG (interaction: group × time). Paired student’s t tests were carried out to evaluate within-group effects. Parametric free tests were used when the requirements for parametric tests were not fulfilled. The association between two continuous variables was proved by the Pearson correlation coefficient. A *p*-value less than 0.05 (two-tailed) was considered statistically significant. Values are presented as mean ± SD. Partial eta squared (η^2^p) was used as an effect size with the classifications small (0.01), medium (0.06), and large (0.14) [24]. Data analyses were conducted with the SPSS statistical software package (Version 24.0, IBM, Armonk, NY, USA).

## 3. Results

Participants of the AG reported an average weekly exercise time of 6.0 ± 2.0 h (predominantly running, cycling, fitness training, hiking, alpine skiing, and ski mountaineering). The SG reported a weekly physical activity time of 2.6 ± 2.0 h (predominantly commuting to work by bike or walking). The participants of the SG completed 82 ± 4% of the supervised training sessions. Missed training sessions were reported to be completed at home without supervision.

### 3.1. Training Adaptions within the SG 

Results are presented in Table 2. Body mass and body composition of the SG remained unaffected by the training. CRF, expressed as VO_2_peak (mL/min and mL/kg/min) and absolute and relative PPO, did not significantly change after the circuit training intervention in the SG. SBP at submaximal exercise significantly (*p* = 0.011) decreased while DBP did not change. During the incremental test, muscle reoxygenation measured by NIRS significantly changed from pre- to post-test (7.0 ± 6.3 vs. 11.0 ± 8.9%), while muscle deoxygenation remained the same. Alterations in muscle reoxygenation were significantly related to changes in BLApeak (r = −710; *p* = 0.032) for SG.

### 3.2. Training Adaptions within the AG

Within the AG, VO_2_peak (mL/kg/min), PPO (watts and watts/kg), BLApeak, HRpeak, and FM significantly decreased over the course of the study while body weight and FFM significantly increased (Table 2). 

### 3.3. Between-Group Differences in Training Adaptions 

No significant differences in changes from pre to post were found between the SG and AG (Table 2).

## 4. Discussion

Results from the present study suggest that the introduction of regular (once per week) circuit training in previously sedentary individuals was not a sufficient stimulus to improve but was able to reduce the expected aging-related decline indices of CRF (VO_2_peak and PPO). It also resulted in reduced SBP during submaximal exercise and improved muscle reoxygenation after exercise. In contrast, CRF indices decreased in the AG over the intervention period. 

We hypothesized that the implementation of one single supervised training session per week would confer increases in CRF by both the session itself and by stimulating participants to increase their physical activity level. In the long term, this was assumed to equalize group differences in CRF. Our data, however, show that this was not the case, even though the exercise training regime slightly reduced the aging-related decrease in CRF. The type of exercise that we implemented and the inability to increase the PA level could be responsible for this negative result. This is supported by the literature, showing that even when performed 2–3 times/week for 20 weeks, different training types that are not specifically focused on improving endurance capacity may have no effect on VO_2_peak in men aged 55–75 years [25]. In contrast, Murias et al. [26] reported significant increases in VO_2_peak in young and old age groups following 12 weeks of training. In that study, improvements were due to increases in maximum cardiac output and maximal arterial-venous oxygen difference. However, training dose (3 times per week for 45 min) and exercise modality (cycling) were different to that in the present study. Similarly, Sillanpää et al. [27], who compared combined high-intensity strength and endurance training with endurance and strength training alone, demonstrated improved VO_2_peak in the combined and the endurance training group in 40–65-year-old men after 21 weeks of training (at least 2 training sessions per week). Thus, we may conclude that cardiorespiratory training effects largely depend on the dose (duration × intensity) of exercise training, which was too low in our study to improve but was sufficient to reduce the ageing-related CRF decline. This is also supported by the results of Burtscher et al. [28] demonstrating that in patients with impaired fasting glucose, at least two supervised training sessions (1.8 h) per week were necessary to maintain exercise capacity and to counteract the aging-related decline. In the AG, both the age-related performance decline (expressed in mL/kg/min), which is greater in endurance-trained compared to sedentary individuals [29], and a slightly reduced training workload may have contributed to the observed CRF decrease.

Regarding the NIRS data, the SG demonstrated significant improvements in restoration of oxygen levels without alterations in oxygen extraction, whereas both reoxygenation and oxygenation did not significantly change in the AG. Ichimura et al. [30] reported that the recovery time of muscle oxygenation is an indicator of aerobic function of the muscle and that after submaximal exercise, recovery time is suggested to predominantly depend on O_2_ delivery to the working muscle. This may by supported by the inverse relation between alterations in muscle reoxygenation and BLApeak, which we found in the present investigation. O_2_-pulse remained the same, indicating unchanged central adaptions [31] and reflecting the unchanged VO_2_peak after the training intervention. The significant reduction in SBP during submaximal exercise (from 180 ± 14 to 170 ± 17 mmHg) in SG may indicate improved tolerance of submaximal exercise, which is of particular importance regarding cardiovascular health as exaggerated BP responses to submaximal exercise are precursors for future cardiovascular diseases [32]. Kjeldsen et al. [33] found that SBP during submaximal exercise was a stronger predictor for cardiovascular mortality and morbidity than BP at rest. However, in the AG, SBP during submaximal exercise did not significantly change over the course of the study. Hence, once weekly circuit training in previously sedentary persons seems to confer peripheral adaptations that may be beneficial for everyday activities (e.g., less lactate production, better recovery) and to have a positive effect on SBP during exercise, indicating a reduced risk for cardiovascular morbidity/mortality and cardiovascular events [34]. 

The present training period did not influence body mass or body composition of the SG. Several studies demonstrated significant weight loss and reduced fat mass after circuit weight training, particular in older obese individuals [8]. With a BMI of 25.6 ± 3.4 kg/m^2^, the participants of the SG were marginally out of the normal weight range, and may benefit less from regular exercise than obese people concerning body mass and body composition. 

The effect of the training intervention on resting SBP was not significant but showed a slight reduction of −3.5 ± 13.3 mmHg for SG. According to a meta-analysis, strength and endurance training reduce SBP by approximately 3 and 1.9 mmHg, respectively [27]. The beneficial effect of training on BP is even higher in hypertensive elderly individuals [35]. The resting BP of the sedentary people in the present study was on average 120 ± 11 mmHg systolic and 84 ± 12 mmHg diastolic and therefore entirely in the normal range. 

Some limitations of the present study have to be acknowledged. The findings were obtained from a relatively small sample size due to dropouts. The training period was discontinuous due to a 10-week home training program, where participants obtained advice for home training and were encouraged to perform at least one self-managed training session per week. Thus, we cannot guarantee that the participants completed the planned home-training sessions. However, as studies on “real-life” training interventions over a long time span are rare, this must be emphasized as a strength of the present study. 

## 5. Conclusions

In contrast to already regularly active individuals, circuit training once per week over 32 weeks slightly prevented the aging-related decline of CRF in previously sedentary subjects and resulted in significant improvements in restoration of the oxygen level in leg muscles after exercise and reduced systolic BP at submaximal exercise. Despite the lack of a significant interaction effect, these findings are encouraging because they are due to relatively little effort (one training session per week), yet also highlight the need for more regular and specific training regimes when the goal is to improve CRF.

## Figures and Tables

**Table 1:** Sex- and group-specific baseline characteristics (MW ± SD) of the participants.

	SG			AG		
	Female (*n* = 5)	Male (*n* = 5)	Total (*n* = 10)	Female (*n* = 3)	Male (*n* = 7)	Total (*n* = 10)
Age [years]	52 ± 5	51 ± 8	51 ± 6	53 ± 11	51 ± 7	51 ± 8
Height [m]	1.72 ± 0.05	1.78 ± 0.04	1.74 ± 0.08	1.65 ± 0.05	1.78 ± 0.05	1.74 ± 0.07
BMI [kg/m^2^]	24.4 ± 2.9	26.8 ± 3.7	25.6 ± 3.1	22.6 ± 0.7	25.2 ± 0.8	24.4 ± 1.4

SG, sedentary group; AG, active group; BMI, body mass index.

**Table 2:** Resting and exercising parameters from pre-to post-training. Values are means ± SD.

	Sedentary Group			Active Group			Group × Time	Time	η^2^p	η^2^p
	*n*	Pre-Test	Post-Test	*n*	Pre-Test	Post-Test	*p*-Value	*p*-Values	Group × Time	Time
Resting parameters										
HRrest [bpm]	9	74 ± 13	78 ± 13	10	66 ± 13	65 ± 10	0.515	0.534	0.025	0.023
Body mass [kg]	10	78.2 ± 10.9	78.5 ± 11.9	10	74.5 ± 9.3	75.6 ± 9.8 *	0.410	0.142	0.038	0.116
SBP [mmHg]	10	120 ± 11	117 ± 15	10	126 ± 13	128 ± 24	0.422	0.905	0.036	0.001
DBP [mmHg]	10	84 ± 12	84 ± 10	10	83 ± 9	88 ± 12	0.306	0.226	0.058	0.080
FFM [%]	10	71.2 ± 8.7	72.6 ± 6.3	10	76.9 ± 4.8	78.2 ± 5.3 *	0.960	0.184	0.000	0.096
MM [%]	10	47.0 ± 6.2	48.0 ± 5.5	10	53.6 ± 4.6	54.2 ± 4.7	0.695	0.128	0.009	0.124
FM [%]	10	27.8 ± 6.7	27.4 ± 6.3	10	23.1 ± 4.8	21.8 ± 5.3 *	0.511	0.222	0.024	0.082
Exercise testing										
VO_2_peak [mL/min]	9	2367 ± 494	2232 ± 461	9	3246 ± 859	3086 ± 795	0.822	0.018	0.003	0.301
VO_2_peak [mL/kg/min]	9	30.8 ± 6.9	28.9 ± 6.0	9	43.1 ± 7.3	40.3 ± 6.5 *	0.509	0.005	0.028	0.405
PPO [watts]	10	181 ± 45	171 ± 44	10	252 ± 66	235 ± 65 *	0.484	0.012	0.028	0.300
PPO [watts/kg]	10	2.3 ± 0.6	2.2 ± 0.6	10	3.3 ± 0.6	3.1 ± 0.6 *	0.258	0.009	0.070	0.321
BLApeak [mmol/L]	10	8.4 ± 2.3	7.3 ± 2.0	10	11.0 ± 2.4	8.8 ± 2.9 *	0.218	0.002	0.083	0.428
HRpeak [bpm]	10	174 ± 10	171 ± 13	9	178 ± 10	172 ± 8	0.563	0.016	0.021	0.314
RPEpeak	10	18.3 ± 1.7	17.4 ± 1.3	10	18.4 ± 1.4	17.4 ± 1.3	0.908	0.063	0.001	0.188
SBP 100 watts [mmHg]	7	180 ± 14	170 ± 17 *	9	173 ± 21	171 ± 18	0.330	0.146	0.068	0.145
DBP 100 watts [mmHg]	7	100 ± 20	93 ± 11	9	89 ± 9	86 ± 10	0.525	0.184	0.029	0.122
HRsubmax [bpm]	9	128 ± 11	125 ± 11	9	113 ± 15	114 ± 16	0.616	0.643	0.016	0.014
BLAsubmax [mmol/L]	9	1.9 ± 0.8	2.1 ± 2.0	10	1.6 ± 0.7	1.7 ± 0.8	0.741	0.412	0.007	0.040
Deoxygenation [%]	9	−9.2 ± 8.3	−8.3 ± 11.3	7	−15.4 ± 7.8	−17.9 ± 9.0	0.267	0.600	0.087	0.020
Reoxygenation [%]	9	7.0 ± 6.3	11.0 ± 8.9 *	7	12.3 ± 8.1	13.6 ± 10.7	0.230	0.028	0.101	0.299
nTHImean [au]	9	0.966 ± 0.127	0.974 ± 0.084	6	1.053 ± 0.118	1.094 ± 0.078	0.613	0.455	0.020	0.044
nTHI30 s [au]	9	0.969 ± 0.187	1.003 ± 0.093	7	1.037 ± 0.149	1.091 ± 0.113	0.802	0.285	0.005	0.081

HRrest, resting heart rate; SBP systolic blood pressure; DBP, diastolic blood pressure; FFM, fat free mass; MM, muscle mass; FM, fat mass; VO_2_peak; peak oxygen uptake; PPO, peak power output; BLApeak, peak blood lactate concentration; HRpeak, peak heart rate; RPEpeak, peak ratings of perceived exertion; SBP 100 watts, systolic blood pressure measured at submaximal exercise (100 watts); DBP 100 watts, diastolic blood pressure measured at submaximal exercise (100 watts), HRsubmax, submaximal heart rate (75 watts for females, 100 watts for males); BLAsubmax, submaximal blood lactate concentration (75 watts for females, 100 watts for males); nTHImean, mean normalized total hemoglobin index during incremental test; nTHI30 s, normalized total hemoglobin index over the last 30 s in the incremental test; * significant within group change (*p* ≤ 0.05); η^2^p, effect size partial η squared.

## Data Availability

Data are contained within the article.

## References

[1] Szostak J., Laurant P. (2011). The forgotten face of regular physical exercise: A ‘natural’ anti-atherogenic activity. Clin. Sci..

[2] Myers J., Prakash M., Froelicher V., Do D., Partington S., Atwood J.E. (2002). Exercise capacity and mortality among men referred for exercise testing. N. Engl. J. Med..

[3] Kokkinos P., Myers J., Kokkinos J.P., Pittaras A., Narayan P., Manolis A., Karasik P., Greenberg M., Papademetriou V., Singh S. (2008). Exercise capacity and mortality in black and white men. Circulation.

[4] Kodama S., Saito K., Tanaka S., Maki M., Yachi Y., Asumi M., Sugawara A., Totsuka K., Shimano H., Ohashi Y. (2009). Cardiorespiratory fitness as a quantitative predictor of all-cause mortality and cardiovascular events in healthy men and women: A meta-analysis. JAMA.

[5] Lee I.M., Shiroma E.J., Lobelo F., Puska P., Blair S.N., Katzmarzyk P.T., Group L.P.A.S.W. (2012). Effect of physical inactivity on major non-communicable diseases worldwide: An analysis of burden of disease and life expectancy. Lancet.

[6] Murias J.M., Kowalchuk J.M., Paterson D.H. (2010). Time course and mechanisms of adaptations in cardiorespiratory fitness with endurance training in older and young men. J. Appl. Physiol..

[7] Chodzko-Zajko W.J., Proctor D.N., Fiatarone Singh M.A., Minson C.T., Nigg C.R., Salem G.J., Skinner J.S., American College of Sports Medicine (2009). Exercise and physical activity for older adults. Med. Sci. Sports Exerc..

[8] Romero-Arenas S., Martínez-Pascual M., Alcaraz P.E. (2013). Impact of resistance circuit training on neuromuscular, cardiorespiratory and body composition adaptations in the elderly. Aging Dis..

[9] Buckley S., Knapp K., Lackie A., Lewry C., Horvey K., Benko C., Trinh J., Butcher S. (2015). Multimodal high-intensity interval training increases muscle function and metabolic performance in females. Appl. Physiol. Nutr. Metab..

[10] McRae G., Payne A., Zelt J.G., Scribbans T.D., Jung M.E., Little J.P., Gurd B.J. (2012). Extremely low volume, whole-body aerobic-resistance training improves aerobic fitness and muscular endurance in females. Appl. Physiol. Nutr. Metab..

[11] Ramos-Campo D.J., Andreu-Caravaca L., Carrasco-Poyatos M., Benito P.J., Rubio-Arias J. (2021). Effects of circuit resistance training on body composition, strength, and cardiorespiratory fitness in middle-aged and older women: A systematic review and meta-analysis. J. Aging Phys. Act..

[12] Myers T.R., Schneider M.G., Schmale M.S., Hazell T.J. (2015). Whole-body aerobic resistance training circuit improves aerobic fitness and muscle strength in sedentary young females. J. Strength Cond. Res..

[13] Kaikkonen H., Yrjämä M., Siljander E., Byman P., Laukkanen R. (2000). The effect of heart rate controlled low resistance circuit weight training and endurance training on maximal aerobic power in sedentary adults. Scand. J. Med. Sci. Sports.

[14] Garber C.E., Blissmer B., Deschenes M.R., Franklin B.A., Lamonte M.J., Lee I.M., Nieman D.C., Swain D.P., American College of Sports Medicine (2011). Quantity and quality of exercise for developing and maintaining cardiorespiratory, musculoskeletal, and neuromotor fitness in apparently healthy adults: Guidance for prescribing exercise. Med. Sci. Sports Exerc..

[15] Warburton D.E., Bredin S.S. (2016). Reflections on physical activity and health: What should we recommend?. Can. J. Cardiol..

[16] Clarke J., Janssen I. (2013). Is the frequency of weekly moderate-to-vigorous physical activity associated with the metabolic syndrome in Canadian adults?. Appl. Physiol. Nutr. Metab..

[17] Glowacki S.P., Martin S.E., Maurer A., Baek W., Green J.S., Crouse S.F. (2004). Effects of resistance, endurance, and concurrent exercise on training outcomes in men. Med. Sci. Sports Exerc..

[18] Sperlich B., Wallmann-Sperlich B., Zinner C., Von Stauffenberg V., Losert H., Holmberg H.C. (2017). Functional high-intensity circuit training improves body composition, peak oxygen uptake, strength, and alters certain dimensions of quality of life in overweight women. Front. Physiol..

[19] Aittasalo M., Livson M., Lusa S., Romo A., Vähä-Ypyä H., Tokola K., Sievänen H., Mänttäri A., Vasankari T. (2017). Moving to business—Changes in physical activity and sedentary behavior after multilevel intervention in small and medium-size workplaces. BMC Public Health.

[20] Carlin A., Perchoux C., Puggina A., Aleksovska K., Buck C., Burns C., Cardon G., Chantal S., Ciarapica D., Condello G. (2017). A life course examination of the physical environmental determinants of physical activity behaviour: A “Determinants of Diet and Physical Activity” (DEDIPAC) umbrella systematic literature review. PLoS ONE.

[21] Bacon A.P., Carter R.E., Ogle E.A., Joyner M.J. (2013). VO2max trainability and high intensity interval training in humans: A meta-analysis. PLoS ONE.

[22] Borg G.A. (1982). Psychophysical bases of perceived exertion. Med. Sci. Sports Exerc..

[23] Boushel R., Langberg H., Olesen J., Gonzales-Alonzo J., Bülow J., Kjaer M. (2001). Monitoring tissue oxygen availability with near infrared spectroscopy (NIRS) in health and disease. Scand. J. Med. Sci. Sports.

[24] Cohen J. (1988). Statistical Power Analysis for the Behavioral Sciences.

[25] Delecluse C., Colman V., Roelants M., Verschueren S., Derave W., Ceux T., Eijnde B.O., Seghers J., Pardaens K., Brumagne S. (2004). Exercise programs for older men: Mode and intensity to induce the highest possible health-related benefits. Prev. Med..

[26] Murias J.M., Kowalchuk J.M., Paterson D.H. (2010). Mechanisms for increases in V^•^O2max with endurance training in older and young women. Med. Sci. Sports Exerc..

[27] Sillanpää E., Häkkinen A., Punnonen K., Häkkinen K., Laaksonen D.E. (2009). Effects of strength and endurance training on metabolic risk factors in healthy 40–65-year-old men. Scand. J. Med. Sci. Sports.

[28] Burtscher M., Gatterer H., Kunczicky H., Brandstätter E., Ulmer H. (2009). Supervised exercise in patients with impaired fasting glucose: Impact on exercise capacity. Clin. J. Sport Med..

[29] Kusy K., Zieliński J. (2014). Aerobic capacity in speed-power athletes aged 20-90 years vs endurance runners and untrained participants. Scand. J. Med. Sci. Sports.

[30] Ichimura S., Murase N., Osada T., Kime R., Homma T., Ueda C., Nagasawa T., Motobe M., Hamaoka T., Katsumura T. (2006). Age and activity status affect muscle reoxygenation time after maximal cycling exercise. Med. Sci. Sports Exerc..

[31] Neary J.P., McKenzie D.C., Bhambhani Y.N. (2002). Effects of short-term endurance training on muscle deoxygenation trends using NIRS. Med. Sci. Sports Exerc..

[32] Carneiro H.A., Song R.J., Lee J., Schwartz B., Vasan R.S., Xanthakis V. (2021). Association of blood pressure and heart rate responses to submaximal exercise with incident heart failure: The framingham heart study. J. Am. Heart Assoc..

[33] Kjeldsen S.E., Mundal R., Sandvik L., Erikssen G., Thaulow E., Erikssen J. (1997). Exercise blood pressure predicts cardiovascular death and myocardial infarction. Blood Press. Monit..

[34] Lovell D.I., Cuneo R., Gass G.C. (2009). Strength training improves submaximum cardiovascular performance in older men. J. Geriatr. Phys. Ther..

[35] Venturelli M., Cè E., Limonta E., Schena F., Caimi B., Carugo S., Veicsteinas A., Esposito F. (2015). Effects of endurance, circuit, and relaxing training on cardiovascular risk factors in hypertensive elderly patients. Age.

